# Incidence of Terson Syndrome in Treated Subarachnoid Hemorrhage in South Korea: a National Health Insurance Database Study

**DOI:** 10.1038/s41598-019-55566-0

**Published:** 2019-12-13

**Authors:** Eun Hee Hong, Mincheol Seong, Hosuck Yeom, Sungyong Choi, Kyu-Sun Choi, Min Ho Kang, Heeyoon Cho, Yong Un Shin

**Affiliations:** 1 Department of Ophthalmology, Hanyang University College of Medicine, Seoul, Korea; 2 Big Data Division, Seoul Metropolitan Government, Seoul, Korea; 3 Department of Neurosurgery, Hanyang University College of Medicine, Seoul, Korea

**Keywords:** Vision disorders, Epidemiology

## Abstract

The aim of this study is to investigate the incidence and mortality of Terson syndrome in patients with treated subarachnoid hemorrhage (SAH) in South Korea. In this nationwide, population-based study, we used the National Health Insurance(NHI) database (2011–2015) to identify patients aged ≥18 years. Newly diagnosed non-traumatic SAH, treated using clipping or coil embolization, were identified, and Terson syndrome was defined as newly diagnosed retinal or vitreous hemorrhage within 3 months of SAH diagnosis. We identified 22,864 patients with treated SAH (tSAH), 196 of whom had Terson syndrome, with the cumulative incidence during 5 years of 0.86% (95% CI: 0.74–0.98): 1.10% (95% CI: 0.88–1.33) in men and 0.71% (95% CI, 0.58–0.85) in women. The cumulative incidence of Terson syndrome in patients aged under 40 was higher than in those aged 40 or over (1.41% vs. 0.81%; p = 0.007). The mortality rate of Terson syndrome in patients with tSAH was not different from that in those without Terson syndrome (4.08% vs. 7.30%; p = 0.089). This was the first nationwide epidemiological study of Terson syndrome using a population-based database. The incidence of Terson syndrome in patients with tSAH was higher in those age under 40 than in those aged 40 or over.

## Introduction

Terson syndrome is characterized by intraocular hemorrhage, such as vitreous, sub-hyaloid, or intraretinal/sub-internal limiting membrane hemorrhage, associated with subarachnoid hemorrhage (SAH)^1^. According to previous studies, Terson syndrome often involves severe SAH, and patients with Terson syndrome have a significantly worse general condition and greater mortality^2–4^. The nationwide incidence of SAH in South Korea is approximately 15 cases per 100,000 person-year^5^, but the incidence of Terson syndrome among the nationwide SAH population has never been investigated.

In several previous single-institution studies, the incidence of Terson syndrome among patients with SAH was 2.5%–40.5%^2,3,6,7^. A single-hospital study from South Korea reported that the incidence of Terson syndrome was 10.6%^8^. Only one systematic review has focused on Terson syndrome incidence^9^. The incidence of Terson syndrome was 13% in three prospective studies, and 3% in six retrospective studies. However, information on nationwide Terson syndrome incidence is lacking. The National Health Insurance (NHI) database in South Korea includes information on the total population and provides big data, including information on disease occurrence and treatment in the entire population. For this reason, the database allows researchers to obtain population-based, epidemiological data about Terson syndrome.

The current study used the NHI database to investigate (1) the incidence of and mortality from Terson syndrome in patients with treated SAH (tSAH), (2) the percentage of patients with tSAH who underwent ocular examination, and (3) the percentage of patients with Terson syndrome who have undergone vitrectomy in South Korea.

## Methods

This study was approved by the Institutional Review Board (IRB) of Hanyang University Guri Hospital, Gyunggi-do, South Korea. The requirement for written informed consent was waived because of the retrospective design (IRB no. 2017–05–013). The research was conducted according to the tenets of the Declaration of Helsinki.

### Database

We used health claims data recorded between 2011 and 2015 in patients aged 18 years or older who were registered in the Health Insurance Review and Assessment (HIRA) service of South Korea. The Korean National Health Insurance Service (KNHIS) is the single national insurance provider in South Korea, providing >97% of the Korean population with compulsory health insurance. The claims reviewed in the HIRA include data regarding diagnoses, procedures, prescription records, demographic information, and direct medical costs. The medical expenses of the Korean population not insured by the KNHIS are covered by the Medical Assistance Program or the Medical Care for Patriots and Veterans Affairs Scheme, and these claims are also reviewed by the HIRA. Therefore, the HIRA database covers the entire Korean population and contains all medical claims made in Korea. Patients in the HIRA are identified by a unique identification number (Korean Resident Registration Number) assigned to each Korean resident at birth; therefore, health care records can be used without any duplications or omissions.

### Definition of terson syndrome

The HIRA database manages claims using the Korean Classification of Disease (KCD), sixth edition, a modified version of the International Classification of Diseases (ICD), 10^th^ edition, adapted for the Korean healthcare system. The diagnosis of Terson syndrome was based on a KCD code. Specifically, we identified treated patients with non-traumatic SAH registered between 2011 and 2015 using the first SAH diagnostic code (I60) combined with the procedure code for clipping (S4641-2) or coil embolization (M1661-2) within the same admission period. Terson syndrome was defined as a diagnosis of retinal hemorrhage (H35.6) or vitreous hemorrhage (H43.1) within the same admission period as first SAH diagnosis, or within 3 months of that diagnosis. Terson syndrome had a 1-year wash-out period. The exclusion criteria were as follows: (1) diagnosis of Terson syndrome in 2010, (2) retinal or vitreous hemorrhage, or history of vitrectomy (S5121-2), intravitreal injection (S5070), or retinal photocoagulation (S5160) during the 1 year before SAH diagnosis, and (3) newly diagnosed ocular pathology, other than Terson syndrome, that causes retinal or vitreous hemorrhage (retinal vessel occlusion, H34; diabetic retinopathy, H36.0, E10.3+ to E14.3+; exudative age-related macular degeneration, H3531.02) after SAH diagnosis.

### Incidence, mortality, ocular examination rate, and treatment methods in patients with Terson syndrome

The incidence of Terson syndrome among nationwide patients with tSAH was calculated by dividing the number of patients who developed Terson syndrome by the total number of patients with non-traumatic SAH. The annual cumulative incidence (%) was calculated, starting on 1 January of each year between 2011 and 2015. The incidence rates of Terson syndrome by age group and sex were also calculated. We further evaluated and compared the difference in incidence according to treatment methods (clipping or coil embolization). If both treatments were performed, the one performed first was used in the calculation. The mortality of Terson syndrome in patients with tSAH was defined as death within 3 months after Terson syndrome diagnosis. Mortality rates were compared among patients with Terson syndrome among tSAH, those without Terson syndrome among tSAH, and all those with tSAH.

We evaluated the percentage of patients who underwent ocular examination among patients with SAH and among those with Terson syndrome. Patients who underwent ocular examination were identified using the procedure code for fundus examination (E6660) between SAH diagnosis and retinal or vitreous hemorrhage diagnosis. To investigate the ophthalmological treatment methods used to treat Terson syndrome, we calculated the percentage of patients who underwent vitrectomy among those with Terson syndrome. These patients were identified using the procedure code for vitrectomy (S5121-2) within 3 months of Terson syndrome diagnosis.

### Statistical analysis

The cumulative incidence (%), with 95% confidence intervals (CIs), were estimated using Poisson distribution. Chi-square analysis was used to compare incidence rates in terms of sex and treatment methods (clip and coil embolization), as well as to compare mortality rates between the sexes. Odds ratios (ORs) with CIs were estimated using logistic regression analysis adjusted for sex, age, and/or year. All two-sided p-values < 0.05 were considered statistically significant. All analyses were conducted using SAS version 9.3 (SAS Inc, Cary, NC).

## Results

**Figure 1:**
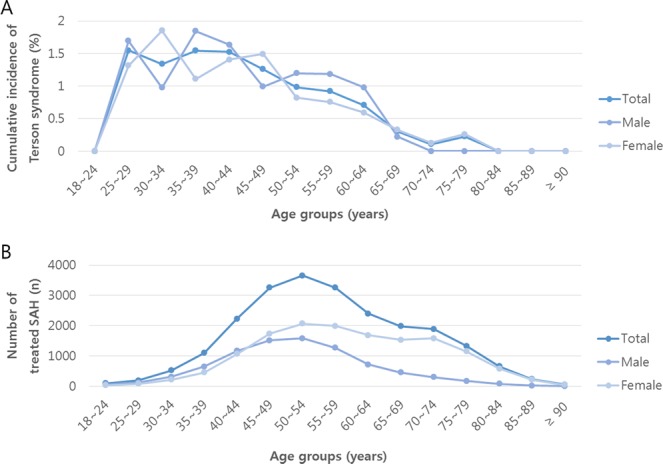
Age distribution of the cumulative incidence of Terson syndrome and number of patients with treated, non-traumatic subarachnoid hemorrhage (SAH) in the Korean population. (**A**) The cumulative incidence of Terson syndrome in treated SAH according to age group. (**B**) Number of patients with treated non-traumatic SAH, according to age groups.

In total, 22,864 treated non-traumatic SAH patients aged 18 years or older (36.8% men) were identified during the 5-year study period (2011–2015). In 2011, 2012, 2013, 2014, and 2015, there were 4,509 (19.7%), 4,444 (19.4%), 4,571 (20.0%), 4,648 (20.3%), and 4,692 (20.5%) treated non-traumatic SAH cases, respectively. Among them, 196 cases of Terson syndrome were identified: 30 (15.3%) in 2011, 30 (15.3%) in 2012, 39 (19.9%) in 2013, 51 (26.0%) in 2014, and 46 (23.5%) in 2015. Table 1 shows the incidences of Terson syndrome in patients with tSAH, according to age group and sex. The cumulative incidence of Terson syndrome in the total population of patients with non-traumatic tSAH during 5 years was 0.86% (95% CI: 0.74–0.98): 1.10% (95% CI: 0.88–1.33) in men and 0.71% (95% CI, 0.58–0.85) in women. Therefore, incidence of Terson syndrome in men was 1.548-times (95% CI: 1.168–2.051) higher than in women (p = 0.004; Table 1). There were no significant differences between each age group, as shown in Table 1. However, patients under 40 years old showed a higher incidence of Terson syndrome than those 40 years of age or older (1.41% vs. 0.81%; p = 0.007). The age distribution of treated non-traumatic SAH differed from that of Terson syndrome: tSAH tended to be more common in middle age, showing distribution pattern that was similar to normal distribution, while Terson syndrome showed a higher incidence in patients under middle age (Fig. 1). The incidence of Terson syndrome showed no significant difference in terms of treatment methods for SAH (clip or coil embolization) (Table 2).

**Table 1:** The cumulative incidence of Terson syndrome in patients with treated subarachnoid hemorrhage in the Korean population from 2011 to 2015 according to age groups and sex.

		tSAH (Person-years)	Terson syndrome (n)	Cumulative incidence (%)	95% CI	OR (95% CI)*	p-value
Age (years)	18–24	95	0	0		13.98 (2.31–84.589)^a^	0.913
	25–29	194	3	1.55	(0–3.28)	12.11 (2.495–58.744)^a^	0.917
	30–34	523	7	1.34	(0.35–2.32)	13.99 (3.208–60.99)^a^	0.913
	35–39	1,101	17	1.54	(0.82–2.27)	13.94 (3.33–58.315)^a^	0.913
	40–44	2,229	34	1.53	(1.02–2.03)	11.56 (2.784–47.976)^a^	0.918
	45–49	3,255	41	1.26	(0.88–1.64)	9.04 (2.169–37.678)^a^	0.924
	50–54	3,658	36	0.98	(0.66–1.30)	8.50 (2.026–35.676)^a^	0.925
	55–59	3,258	30	0.92	(0.59–1.25)	6.59 (1.521–28.594)^a^	0.931
	60–64	2,403	17	0.71	(0.37–1.04)	2.83 (0.57–14.041)^a^	0.951
	65–69	1,986	6	0.30	(0.06–0.54)	1	Reference
	70–74	1,886	2	0.11	(0–0.25)	2.141 (0.357–12.829)^a^	0.958
	75–79	1,328	3	0.23	(0–0.48)	NE	
	80–84	661	0	0		NE	
	85–89	235	0	0		NE	
	90+	52	0	0			
Sex	Male	8,424 (36.8%)	93 (47.4%)	1.10	(0.88–1.33)	1.548 (1.168–2.051)^b^	0.004^†^
	Female	14,440 (63.2%)	103 (52.6%)	0.71	(0.58–0.85)	1	Reference
Total		22,864	196	0.86	(0.74–0.98)	13.979 (2.31–84.589)	0.9132

CI = confidence interval; NA = not applicable; NE = no estimate; OR = odds ratio; tSAH = treated subarachnoid hemorrhage.

^*^OR was estimated using sex-adjusted logistic regression analysis.

^a^OR adjusted for sex and year; ^b^OR adjusted for age and year; ^†^Chi-square test.

**Table 2:** The cumulative incidence of Terson syndrome in patients with treated subarachnoid hemorrhage (SAH) in the Korean population from 2011 to 2015 according to the treatment methods for SAH.

	Clip				Coil embolization				Clip vs. Coil (p-value)*
	tSAH (Person-years)	Terson syndrome (n)	Cumulative incidence (%)	95% CI	tSAH (Person-years)	Terson syndrome (n)	Cumulative incidence (%)	95% CI	
**Sex**									
Male	4,530	57	1.26	(0.93–1.89)	3,894	36	0.93	(0.62, 1.18)	0.148
Female	7,470	42	0.56	(0.39–1.00)	6,970	61	0.88	(0.66, 1.11)	0.027
**Overall**	12000	99	**0**.**83**	(0.66–1.16)	10,864	97	**0**.**89**	(0.72, 1.18)	0.582

CI = confidence interval; OR = odds ratio; tSAH = treated subarachnoid hemorrhage.

*Chi-square test.

**Figure 2:**
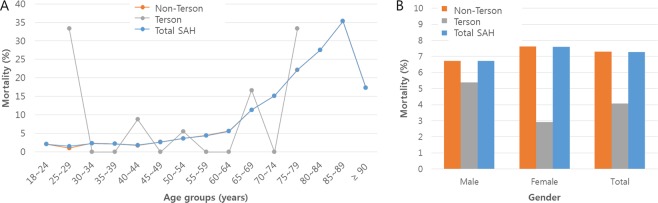
Mortality rates in patients with treated subarachnoid hemorrhage (SAH) with Terson syndrome and in those with treated SAH without Terson syndrome (non-Terson) in the Korean population, according to age and sex. (**A**) Mortality rates in Terson syndrome, non-Terson, and total treated SAH (total SAH) according to age groups. (**B**) Mortality rates in Terson syndrome, non-Terson, and treated SAH (total SAH), according to the sex.

The mortality rate of Terson syndrome was 4.08% over the 5-year study period, and there was no significant difference in mortality rate between the sexes (Table 3). The age distribution of mortality rates in patients with Terson syndrome, in those without Terson syndrome (non-Terson), and in all patients with tSAH (total SAH) are shown in Fig. 2A. The age distribution pattern of mortality rate among patients with Terson syndrome differed from that among those without Terson syndrome (non-Terson) and all patients (total SAH). In the latter two groups, mortality tended to increase with age. There was no significant difference in mortality rates between patients with Terson syndrome and those without (4.08% and 7.30%, respectively; p* = *0.0889) (Fig. 2).

**Table 3:** Mortality rates of Terson syndrome in patients with treated subarachnoid hemorrhage in the Korean population from 2011 to 2015.

	Total N	Death	Mortality	OR (95% CI)	p-value	Adj OR (95% CI)*	p-value^†^
**Sex**							
Male	93	5	5.38	1.894 (0.44–8.153)	0.3912	5.037 (0.475–53.451)	0.1797
Female	103	3	2.91	1	Reference	1	Reference
**Overall**	196	8	4.08				

CI = confidence interval; OR = odds ratio.

^*^OR estimated using age- and year-adjusted logistic regression analysis.

^†^Chi-square test.

**Figure 3:**
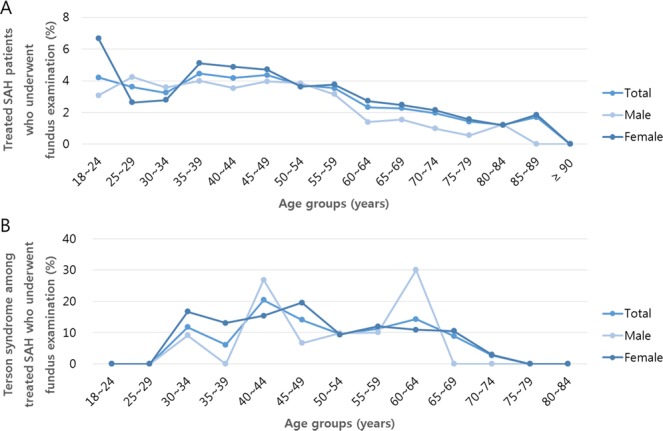
The percentage of patients who underwent fundus examination among those with treated subarachnoid hemorrhage (SAH) and those with Terson syndrome. (**A**) The percentage of patients who underwent fundus examination among those with treated SAH (tSAH). (**B**) The percentage of patients who underwent fundus examination among those with Terson syndrome.

Figure 3 shows the percentage of patients with the procedure code for fundus examination in tSAH and Terson syndrome. Among all patients with tSAH, 3.2% had the procedure code for fundus examination: 3.2% in men and 3.2% in women (Fig. 3A). Among patients with Terson syndrodme, 11.3% of all patients had the procedure code for fundus examination—10.8% of men and 11.6% of women (Fig. 3B). Among patients with Terson syndrome, 36.22% underwent vitrectomy: 46.24% in men and 27.18% in women (Table 4).

**Table 4:** Percentage of patients with Terson syndrome who underwent vitrectomy by age group.

		Total			Male			Female		
		Terson syndrome	Treatment	%	Terson syndrome	Treatment	%	Terson syndrome	Treatment	%
Year	2011	30	14	46.67	20	11	55	10	11	110
	2012	30	12	40	12	6	50	18	6	33.33
	2013	39	9	23.08	21	6	28.57	18	6	33.33
	2014	51	20	39.22	22	9	40.91	29	9	31.03
	2015	46	16	34.78	18	11	61.11	28	11	39.29
Age (years)	18–24	0	0		0	0		0	0	
	25–29	3	1	33.33	2	1	50	1	0	0
	30–34	7	1	14.29	3	1	33.33	4	0	0
	35–39	17	7	41.18	12	5	41.67	5	2	40
	40–44	34	19	55.88	19	12	63.16	15	7	46.67
	45–49	41	14	34.15	15	7	46.67	26	7	26.92
	50–54	36	13	36.11	19	9	47.37	17	4	23.53
	55–59	30	10	33.33	15	6	40	15	4	26.67
	60–64	17	3	17.65	7	1	14.29	10	2	20
	65–69	6	1	16.67	1	1	100	5	0	0
	70–74	2	1	50	0	0		2	1	50
	75–79	3	1	33.33	0	0		3	1	33.33
	80–84	0	0		0	0		0	0	
	85–89	0	0		0	0		0	0	
	90+	0	0		0	0		0	0	
	**Total**	196	71	**36**.**22**	93	43	**46**.**24**	103	28	**27**.**18**

## Discussion

The present study was the first nationwide, population-based epidemiological study of Terson syndrome. To our knowledge, it was the largest study of its kind. Our results provide informative data regarding the incidence, mortality, and treatment methods of Terson syndrome among patients with treated, non-traumatic SAH in the South Korean population aged ≥18 years. The data were gathered over a 5-year period. In South Korea, the incidence of Terson syndrome among patients with treated, non-traumatic SAH was 0.86%, and the incidence was higher in men than in women (1.10% vs. 0.71%; OR = 1.548, p = 0.004). The mortality rate of Terson syndrome in patients with tSAH was not higher than that in patients with tSAH without Terson syndrome (4.08% vs. 7.30%).

The average incidence of Terson syndrome in patients with tSAH in the present study was lower than in previous reports. In previous retrospective studies conducted in single institutions, the reported incidence of Terson syndrome varied. In one such retrospective report, Lee *et al*. reported that the incidence of Terson syndrome in a Korean hospital between 2007 and 2013 was 10.6% (34 of 332 patients with aneurysmal SAH)^8^, while Seif *et al*. reported that the incidence of Terson syndrome in a Canadian hospital from 2012 to 2013 was 21.7% (10 of 46 patients with spontaneous SAH)^3^. With regards to prospective reports, Sung *et al*. reported that the incidence of Terson syndrome in a Brazilian hospital was 29% (14 of 47 patients with spontaneous SAH)^2^, and He *et al*. reported that the incidence of Terson syndrome in a Chinese hospital from 2009 to 2010 was 14.8% (15 of 101 patients with aneurysmal SAH)^7^. One systematic review published in 2004 identified six retrospective studies and three prospective studies that satisfied their inclusion criteria^9^. That review reported that the mean incidence in retrospective studies was 3% (37/1086), while that among all prospective studies was 13% (24/181), so the prospective studies showed a higher frequency of Terson syndrome than the retrospective studies, suggesting that vitreous or retinal hemorrhage is not well documented. However, the prospective studies from the review were all published in the 1990s, while the retrospective studies were conducted in the 1970s and 1980s. The present study used health claims data, so the incidence of Terson syndrome may have been underestimated, because asymptomatic cases or patients for whom the diagnosis was not registered in the database could not be identified using health claims data. Therefore, the incidence rates reported in the present study only pertained to clinically diagnosed Terson syndrome. Furthermore, the incidence of Terson syndrome may have been relatively low in the present study because we excluded other retinal disorders that can cause retinal or vitreous hemorrhage, such as retinal vessel occlusion, diabetic retinopathy, and exudative age-related macular degeneration. These disorders can be accompanied by Terson syndrome, but the cause of retinal or vitreous hemorrhage cannot be distinguished based on diagnostic code alone. In addition, including only patients with treated SAH might be another possible reason for this underestimation. Among patients with non-treated SAH who did not require a treatment because their case was mild or could not afford a treatment due to it being too severe, Terson syndrome may also occur. Although we cannot determine whether Terson syndrome among non-treated SAH would increase or decrease the total incidence of Terson syndrome, this will cause measurement bias.

With regards to sex distribution, the present study showed a higher incidence of Terson syndrome in men than in women. Previous studies have reported that both Terson syndrome and SAH are more common in women than in men^2,3,9–12^. In the present study, about 1.7-times more women than men were diagnosed with tSAH (14,440 vs. 8,424), while 1.1-times more women than men were diagnosed with Terson syndrome (103 vs. 93), consistent with previous studies. It seems that the incidence of Terson syndrome, calculated as the number of patients diagnosed with Terson syndrome divided by the number of patients with tSAH, was lower in women than in men because the women-to-men ratio was higher in the denominator than in numerator (1.7-times vs. 1.1-times). Previous studies have shown that women are at greater risk of aneurysmal SAH than men^10–12^, and that they have a greater likelihood of developing multiple aneurysms^13^. Regarding the reason of sex predilection of aneurysm formation, it has been postulated that women have weaker vessel walls than men, which may explain the sex-associated predilection for aneurysm formation. Furthermore, differences in collagen and elastin interference factors, as well as hormonal changes, may contribute to aneurysm formation^14^. Although several reports have shown that more women than men have Terson syndrome, these reports compared the *number* of patients with Terson syndrome according to the sex, rather than the *incidence* of Terson syndrome^2,3^. It remains unclear why gender differences are present in Terson syndrome.

Several previous studies have reported the mean age in SAH and Terson syndrome cases. Seif *et al*. reported that the mean age of patients with both SAH and Terson syndrome was around 60 years^3^, while Sung *et al*. reported that it was around 49 years^2^. Lee *et al*. reported that the mean age of patients with both SAH and Terson syndrome was 54.3 years, while that of patients with SAH and without Terson syndrome was 56.0 years^8^. However, no studies have described the age distribution of patients with Terson syndrome. In the present study, there was a higher incidence of Terson syndrome in patients under 40 years old than in those 40 years of age or older. In addition, the age distribution of patients with Terson syndrome differed from that of patients with tSAH in a pattern similar to normal distribution, as shown in Fig. 1.

Next, we further investigated the incidence rates of Terson syndrome depending on the methods used to treat SAH (clip or coil embolization). Lee *et al*. reported that, in aneurysmal SAH, the treatment method differed between patients with Terson syndrome and those without the syndome; endovascular coil embolization was significantly correlated with Terson syndrome diagnosis^8^. They hypothesized that cerebrospinal fluid release during microsurgical aneurysmal clipping may prevent increases in intracranial pressure, and that this may influence the occurrence of intraocular hemorrhage. In the present study, the overall incidence of Terson syndrome showed no significant difference between treatment types for SAH. However, in females, the incidence rate of Terson syndrome was higher in the coil embolization group, and the reason for this is unclear. Factors known to potentially be related to the occurrence of Terson syndrome, including the poor neurological condition of SAH^7,15^, a history of transient or prolonged coma before admission^4,16–18^, increased intracranial pressure^18^, and location, laterality, and size of aneurysms^19^, could not be investigated with the health claims data presented here. In order to determine whether the higher incidence in the coil embolization group in females is a meaningful result, these confounding factors need to be adjusted. Furthermore, the size and location of aneurysms also affect treatment methods in patients with aneurysmal SAH. A more detailed review of medical records will be necessary to elucidate the relationship between Terson syndrome and the methods used to treat SAH, as well as the relationship with sex.

In the present study, the mortality rate of Terson syndrome was 4.08% during the 5-year study period. It was higher in men than in women (5.38% vs. 2.91%) but with no significance difference (p = 0.1797). Moreover, the mortality rate of tSAH showed no significance difference according to the presence of Terson syndrome (4.08% in Terson syndrome vs. 7.30% in non-Terson syndrome; p = 0.0889). Previous studies based only on hospital data have reported that patients with Terson syndrome have a significantly worse general condition and greater mortality^2,3,9^. Seif *et al*. reported that patients with Terson syndrome have significantly worse Glasgow Coma Scale (GCS) and Hunt/Hess scores upon admission to the hospital, as well as lower modified Rankin scores (a measure of neurological morbidly) upon discharge, and greater mortality^3^. In a prospective report by Sung *et al*., the presence of Terson syndrome was associated with an increased mortality rate (50% vs. 9%; p < 0.01)^2^. He *et al*. reported a higher frequency of Terson syndrome in patients with consciousness disturbance, lower GCS score, and higher Hunt-Hess grade, but no correlation between mortality rate and Terson syndrome^7^. According to a systematic review by McCarron *et al*.^9^, deaths were recorded in five studies, and that 50% of patients with Terson syndrome (12 of 24) died, compared to 11% of SAH patients without Terson syndrome (17 of 157) in three prospective studies. They also reported that 17% of patients with Terson syndrome (1 of 6) died, compared to 8% of SAH patients without Terson syndrome (14 of 185) in two retrospective studies. The overall mortality rate was 43% (13 of 30) in patients with Terson syndrome, compared with 9% of SAH patients without Terson syndrome (31 of 342), giving a 4.8-fold excess risk of death in patients with Terson syndrome (p < 0.001). Retrospective studies, including the present study, may have underestimated the mortality rate of Terson syndrome, because patients who die are unlikely to have had good enough general condition to undergo eye examination. The inconsistency between the present and previous studies may also have been due to differences in the definition of mortality. Specifically, we defined the mortality of Terson syndrome in patients with tSAH as death within 3 months of Terson syndrome diagnosis, excluding possible confounding factors that can affect death. Thus, we may have underestimated the mortality rate of Terson syndrome. Furthermore, as this study included only treated SAH, patients with Terson syndrome among non-treated SAH could not be included. Diagnoses of Terson syndrome among patients with severe SAH who could not afford a treatment or with a mild form of SAH who did not require a treatment could not be included according to this study design. Therefore, missing the death of a Terson syndrome with severe non-treated SAH may result in underestimating the mortality of Terson syndrome. On the other hand, including only treated SAH patients may result in an underestimation of mild SAH without Terson syndrome, which leads to an over evaluation of the mortality in non-Terson syndrome.

So far, no population-based reports have ascertained the rate of eye examination in patients with SAH. In the present study, the percentage of patients with tSAH with the procedure code for fundus examination was estimated as 3.2%. This may explain the relatively low incidence of Terson syndrome in this population-based study. Furthermore, the present results suggest that the rate of patients with SAH who underwent ocular examination was very low, that ocular examinations which were actually performed were not registered as the correct procedure code properly, or both. Firstly, patients with poor general condition or with other co-morbidities usually require bed-side examination, and these patients have a higher frequency of missing procedure codes for fundus examination even though the examination has been performed. Indeed, the percentage of patients with Terson syndrome among those with tSAH who underwent ocular examination was 11.34% (not 100%), indicating that the procedure codes were poorly registered when the ocular examination was performed, because Terson syndrome cannot be diagnosed without ocular examination. Therefore, the rate of eye examination in this study should be interpreted as the rate at which the procedure code of “fundus examination” was registered, not as the actual performance rate. In addition, Table 4 and Fig. 3 show that in age groups where the percentage of patients underwent PPV is high, the percentage of patients who underwent fundus examination is also high. This suggests that the registration rate of the procedure code for fundus examination appears to be relatively high if active ophthalmological treatment is undertaken, which is also a limitation of the use of health claims data. Secondly, from the point of view of actual performance of fundus examination, patients with severe SAH were unable to undergo eye examination due to their poor general condition. In addition, even if Terson syndrome occurs in patients with tSAH, the eye examination may not be performed unless they report visual symptoms. In the elderly population, where there may be age-related difficulties in identifying visual symptoms or decreased visual acuity caused by age-related disorders such as cataracts or macular degeneration (dry type), the actual performance rate of eye examination may also be low.

In addition, no previous studies have evaluated the methods used to treat Terson syndrome. In the present study, the percentage of patients with Terson syndrome who underwent vitrectomy within 3 months of diagnosis was 36.2%, indicating that the majority of patients with Terson syndrome recover without ocular surgery, but also that one-third of them require surgery.

There were several limitations in the present study. Firstly, it was a population-based study, so the incidence may have been underestimated. We may also have overestimated the number of patients with SAH, as determined from health claims data. For this reason, we limited the definition of SAH to “treated SAH”. This might have also led to a rather large difference in the incidence or mortality of Terson syndromes shown in previous studies. Therefore, we limited the subjects of the study to Terson syndrome in patients with treated SAH. Furthermore, we could not identify asymptomatic cases, or patients for whom the diagnosis was not registered in the database, so the number of patients with Terson syndrome may have been underestimated. Accordingly, our results only pertained to the incidence rate of clinically diagnosed Terson syndrome. Secondly, prevalent cases of SAH or intraocular hemorrhage may still have been included, although we used a 1-year period to infer disease-free status. Thirdly, by using the health claims database, all of the data we used were based on the registration of the procedure or diagnostic code. If the examiner did not register the diagnostic code or procedure code, the health insurance data could not identify this. It is a huge advantage of using health insurance data to analyze the data of the entire population, but such limitations should be taken into account.

In conclusion, we have carried out the first incidence study of Terson syndrome in South Korea using a population-based database over 5 years. The incidence of Terson syndrome in patients with tSAH was higher in men than in women, and it was higher in patients under 40 years old than in those 40 years of age or older. The mortality rate in patients with Terson syndrome among those with tSAH was not higher than that in patients with tSAH without Terson syndrome. The results of this study suggests that patients with SAH tend to undergo eye examinations in the presence of visual symptoms, rather than receiving eye examinations to screen for Terson syndrome. Considering the legal issue of visual impairment due to Terson syndrome, and the importance of identifying the possible causes of visual problems in patients with SAH, clinicians should perform fundus examination when patients with SAH in cooperative conditions complain of visual. This information may provide insight regarding the incidence and mortality of Terson syndrome, as well as ocular examination patterns in patients with tSAH in South Korea.

## Data Availability

The data that support the findings of this study are available from the corresponding author upon reasonable request.
